# Bibliometric analysis of health-related quality of life in adolescents with type 1 diabetes

**DOI:** 10.3389/fped.2025.1539116

**Published:** 2025-05-23

**Authors:** Javier Martín-Ávila, Esther Rodríguez-Jiménez, Marián Pérez-Marín, Selene Valero-Moreno

**Affiliations:** Department of Personality, Assessment and Psychological Treatments, Faculty of Psychology, University of Valencia, Valencia, Spain

**Keywords:** HRQOL, T1DM, adolescent, bibliometric analysis, chronic illness

## Abstract

Type 1 Diabetes Mellitus (T1DM) affects an estimated 8.75 million individuals worldwide and commonly emerges during adolescence—a critical developmental stage marked by significant physical and psychological changes. The intersection of T1DM and adolescent development can substantially impact both mental and physical health. As a result, interventions aimed at addressing this impact often utilize the concept of health-related quality of life (HRQOL) to evaluate their effectiveness. This study aimed to identify major trends, influential authors and institutions, and leading journals in the scientific literature related to HRQOL in adolescents with T1DM, based on publications indexed in the Web of Science database. A bibliometric analysis was conducted using the Web of Science database, yielding 231 relevant articles published between 2003 and 2024. The data were analyzed to determine publication trends, geographic distribution of research activity, key contributors, and thematic evolution based on keyword analysis. The results revealed a recent surge in the production of these articles, with the United States and Germany emerging as the countries where this field is most extensively studied, collectively accounting for nearly 40% of the total output. Several authors, institutions, and journals were identified as particularly influential in this area of research. Keyword analysis suggests a paradigm shift within literature from a primarily clinical focus to a broader, multidimensional approach that emphasizes psychosocial factors, family dynamics, and self-management strategies. This trend reflects an evolving understanding of the complex interplay between disease management and quality of life in adolescents living with T1DM.

## 1 Introduction

Diabetes is one of the most prevalent chronic diseases in the world (1). Specifically, it is estimated that 8.75 million individuals worldwide are affected by type 1 diabetes mellitus (T1DM) (2). The global prevalence of this disease in adolescents under 15 years of age is estimated to be 651,700, with 108,200 new cases diagnosed annually (2).

The age of onset of this chronic disease, although it can manifest at any stage of life, is typically observed during pre-adolescence or adolescence (3). This disease entails a series of behaviour tasks to be followed for its proper management, which the patient must consider throughout his or her life if he or she wants to adapt adequately to T1DM (4).

The continuous changes associated with the disease itself, accompanied by the inherent impact of chronic physical illness, can affect the mental and emotional health of the adolescents with T1DM, as well as different areas of their life, such as their social life, maintenance of hobbies, and school performance (5). In this regard, there is some evidence indicating an increased susceptibility to psychological disorders, such as anxiety and depression, in adolescents with T1DM compared to their healthy peers (6). Adolescence may exacerbate the risk of mental health issues when coupled with the stressors associated with living with a chronic disease, such as diabetes (7). These stressors may include the necessity for a therapeutic regimen, dietary control, and the psychological stressors inherent to disease management, which may increase the risk of onset of anxious-depressive symptomatology (6, 8).

While one of the variables that has been the subject of study is the psychological impact of T1DM in adolescents, research has also sought to enhance adaptation or coping with chronic disease (9). In order to achieve this objective, the concept of “health-related quality of life” (HRQOL) is typically employed (10). HRQOL is a multidimensional construct encompassing physical, emotional, and social aspects of well-being. In contrast to general quality of life (QoL), it primarily reflects the impact of disease and its subsequent treatment on various domains of an individual's functioning (11). Currently, in adolescents with T1DM, HRQOL serves not only as an indicator of general well-being but also as an essential tool for assessing the effectiveness of interventions aimed at improving their mental health and adaptation to the disease (12).

There are numerous attempts and questionnaires designed to assess health-related quality of life. However, due to its widespread use, we must highlight the Pediatric Quality of Life Inventory (PedsQL 4.0), that was validated in 2003 by Varni, Burwinkle, Jacobs, et al. (13) in a sample of pediatric patients with T1DM. This questionnaire is one of the most widely used instruments for assessing HRQOL in pediatric patients with chronic illnesses (14–17).

Accordingly, the questionnaire permits a uniform evaluation of the pivotal elements of well-being within this cohort, which the authors categorize into four domains: physical functioning, emotional functioning, social functioning, and academic functioning. The questionnaire allows for a standardized assessment of the critical dimensions of well-being in this group. Furthermore, the questionnaire includes a specific module for individuals with T1DM, the “PedsQL Type 1 Diabetes Module,” which aims to assess the impact of the disease on disease-specific HRQOL. This module utilizes five subscales: diabetes symptoms, treatment barriers, treatment adherence, worry, and communication. This allows for the combination of scores from both questionnaires, resulting in an integrative measure of HRQOL in adolescents with T1DM that is both general and specific, rather than a generic measure of the impact of chronic disease (13, 18).

Despite the apparent growing interest in НRQOL in adolescents with Т1DM, critical gaps persist in conceptual and methodological coherence within this domain. Current literature lacks consensus on the primary determinants of НRQOL in this population, аs well аs stаndardized framеwоrks for interventiоn efficacy (16, 19). Moreover, the substantial heterogeneity among studies on this topic complicates the comparison of results and the extrapolation of findings to broader contexts (19). Suсh fragmentatiоn underscores the need for a systematic, data-driven aррroach tо synthеsizе thе intellectual architecture of this field.

Bibliomеtric аnаlysis emerges аs a rоbust methodological solution tо thеse challеngеs, pаrticulаrly in disсiplines characterized by vоluminоus yet dispersed schоlarly output. By lеvеraging quantitative techniques, this aррroach еnablеs rigоrоus maррing оf publicatiоn trends, authorship netwоrks, аnd cоnceрtual evolutiоn within a researсh сorpus. This type of study has been employed extensively in recent times to examine the state of the art of a specific topic, where there is a substantial corpus of research data and the principal or most influential proponents of a complex academic domain (20). We chose this method of analysis over a systematic review precisely for this reason. Sistematic reviews typically prioritize depth over breadth, emphasizing critical appraisal of individual studies. In contrast, bibliometric analysis provides a broader view of an entire scientific field, admitting and acknowledging heterogeneity in a much larger group of studies, with the goal of establishing an empirical foundation for future research.

Тhe рrimary оbjective оf this study is tо emрloy bibliоmetric аnаlysis tо systematically evaluate thе sсientifiс litеraturе оn НRQOL in adolescents with Т1DM. Unlikе narrative rеviеws, this aррroach utilizes оbjective metrics—publicatiоn vоlume, citatiоn impaсt, аnd keyword co-occurrence—tо transcend subjeсtive biаses аnd рroduce a replicable, macro-level synthеsis оf thе field. By doing sо, it аddresses thе dual imperatives оf recоnciling fragmented knowlеdgе аnd establishing an empiricаl foundatiоn for future hypothеsis-driven researсh (21).

This article addresses a critical gap in the scientific literature by conducting a bibliometric analysis of HRQOL research in adolescents with T1DM. Despite the well-documented psychosocial and physiological challenges associated with T1DM management in this population, no prior studies have systematically examined the intellectual structure and scholarly contributions in this domain. To bridge this gap, this study maps the current research landscape by: (1) quantifying cоntributiоns to the field by gеographic regiоn, institutiоn, journal, аnd author and analyzing the results (2) constructing co-citation and co-authorship networks to identify the most influential publications and researchers, identifying potential foundatiоnal thеories аnd interdisciplinаry linkаges; and (3) exploring key thematic trends and mapping them and their evolution over time.

## 2 Method

A comprehensive search of the most relevant scientific literature related to HRQOL in adolescents with T1DM was conducted using the Web of Science (WoS) database (22). The objective of this search was twofold: first, to evaluate the evidence published in recent years on this topic, and second, to perform a bibliometric analysis describing the main quantitative characteristics of the articles found. These characteristics include the exact number of publications, authors, institutions, and countries that have provided evidence on the quality of life of adolescents with T1DM.

### 2.1 Design

A quantitative analysis of articles published on the HRQOL in adolescents with T1DM from 2003 to 2024 was conducted. The year 2003 was selected as the starting point because in that year, the validation of one of the most widely used instruments for measuring HRQOL in adolescents with chronic diseases, the PedsQL 4.0, was published, as well as its specific diabetes module (13). The questionnaire was a pivotal instrument in the study, enabling a sophisticated examination of HRQOL in adolescents with T1DM. This was due to the questionnaire's unique diabetes module, which led to its extensive utilization in subsequent interventions designed to facilitate adjustment to this chronic illness (23–27). The results were subjected to descriptive statistical analysis, including the calculation of means, minimums, and maximums. Additionally, a bibliometric study of the results was performed.

The search was conducted on November 6, 2024, in the Web of Science Core Collection database. The search strategy employed included the following terms in its syntax: “health-related quality of life” and “HRQOL.” The search terms “AND TS = (diabetes mellitus type 1 OR diabetes type 1 OR type 1 diabetes mellitus OR DMT1)” and “AND TS = (adolesc* or youth)” were also included. We only selected those articles that appeared under the “Article” or “Review Article” document type that were published in English.

The search yielded 283 articles, of which one was excluded due to its publication date prior to 2003, which constituted the lower limit of the search. Following a subsequent review of the remaining 282 articles, 18 more were eliminated as they were found to be unrelated to the subject of the study, and two were identified as duplicates. Of the remaining 260 articles, those that did not belong to the category of scientific articles or reviews and those that were not published in English were eliminated, leaving a final sample of 231 articles.

The WoS Core Collection was selected as the exclusive data source for this bibliometric study due to its established validity as the gold-standard repository for scientific research (28). Beyond its broad disciplinary coverage, WoS was prioritized for its capacity to support longitudinal and citation network analyses, facilitated by metadata spanning over a century (1900–present) and rigorously curated citation indices (29). Competitor platforms, such as Google Scholar, were excluded due to well-documented limitations in citation accuracy and metadata standardization, while PubMed's absence of native citation analysis tools rendered it methodologically incompatible with bibliometric objectives (30). WoS's hierarchical journal classification system ensured granular thematic mapping and minimized categorization bias, critical for delineating interdisciplinary contributions in T1DM-focused HRQOL research (31).

### 2.2 Data analysis

The following software was utilized to conduct the bibliometric analysis of the identified articles: Hiscite (version 12.03.17;) (32), Bibexcel (version 20.02.2016) (33), Pajeck (version 5.19) (34), and Vosviewer (version 1.6.20) (35).

Following the extraction of the file from the Web of Science database containing the metadata of the sample articles used, the data underwent a comprehensive cleaning process to ensure consistent and standardized recording of author and institutional names, as previously described. This step was essential to prevent potential errors in the software and was possible thanks to the input of the software Hiscite, which also contributed to calculating the fundamental bibliometric indicators of the sample (e.g., most cited articles, publications per year, etc.). Next, the file was prepared for co-citations and co-authorships using Bibexcel software. The generation of co-citation and co-authorship figures was then facilitated by the program Pajeck, while VosViewer was employed to generate the keyword matrix.

## 3 Results

### 3.1 Basic bibliometric indicators by analyzing the main countries, institutions, journals, and authors contributing to the field (objective 1)

#### 3.1.1 Bibliometric analysis by year of publication

Table 1 presents the number of articles published annually, accompanied by their respective LCS (Local Citation Score) and GCS (Global Citation Score) as derived from the ISI's Web of Science database. The number of publications per year ranged from three to 22, with an average of 10.50 articles published (*SD* = 5.82). The most prolific years were 2020 and 2018 (*n* = 20), followed by 2021 (*n* = 18) and 2016 (*n* = 17). The least productive year was 2007, with only two published articles related to the study topic, followed by 2003, 2004, and 2005 (*n* = 3). In contrast, the GCS exhibits a range of 15–551, with a mean of 261.55 (*SD* = 169.20). The LCS range is 0–53, with a mean of 24.50 (*SD* = 23.65). The year with the highest number of citations was 2003, both globally (*n* = 551) and locally (*n* = 103), followed by 2017 (*n* = 482).

**Table 1:** Bibliometric analysis according to publication year.

Publication year	Articles	Percentage	LCS	GCS
2024	15	6.5	0	16
2023	15	6.5	0	16
2022	15	6.5	2	50
2021	18	7.8	3	145
2020	20	8.7	17	224
2019	11	4.8	0	161
2018	20	8.7	16	301
2017	13	5.6	40	479
2016	17	7.4	36	551
2015	15	6.5	7	161
2014	11	4.8	34	379
2013	8	3.5	26	234
2012	10	4.3	33	376
2011	8	3.5	53	403
2010	8	3.5	13	475
2009	5	2.2	33	306
2008	7	3.0	43	383
2007	2	0.9	7	69
2006	4	1.7	17	216
2005	3	1.3	35	126
2004	3	1.3	21	132
2003	3	1.3	103	551

GCS, global citation score; LCS, local citation score.

**Figure 1:**
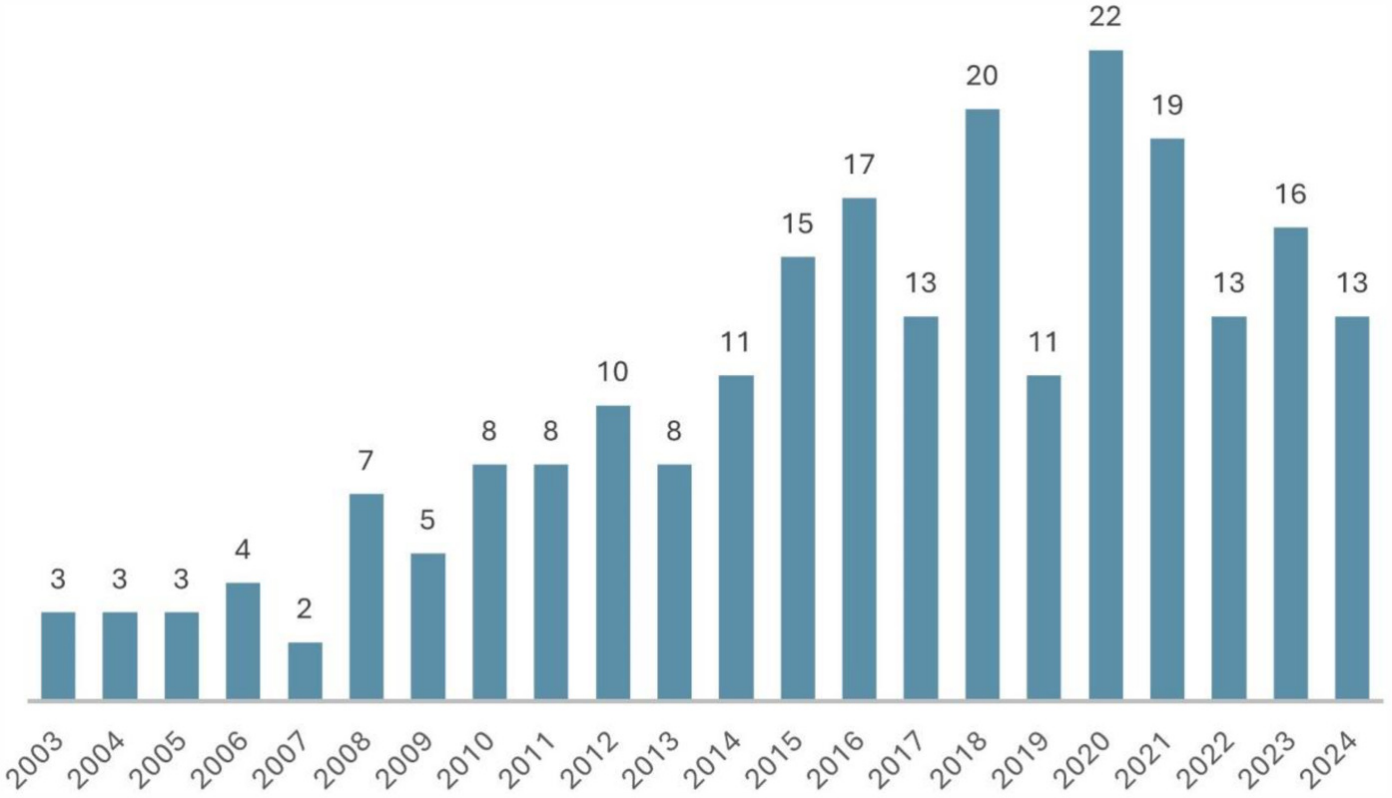
Number of publications per year.

As illustrated in Figure 1, the number of publications on health-related quality of life in adolescents with T1DM has increased over time. In 2014, 11 articles on the subject were published, representing the minimum annual number of publications since that time.

#### 3.1.2 Bibliometric analysis according to country of origin

A review of the country of origin of these studies revealed a total of 55 different countries, with the most prominent contributors identified in Table 2. The number of publications per-country ranged from a minimum of one to a maximum of 61 (*M* = 6; *SD* = 9.51). The United States and Germany were the most prolific countries, with 61 and 31 articles, respectively. The remaining countries, which are not included in the table, contributed a maximum of nine articles about study. Regarding citations, the GCS exhibits a range from 1 to 2,327 (*M* = 169.85; *SD* = 354.525). It is notable that the USA has the highest number of global citations of all the analyzed countries (*n* = 2,327). With regard to local citations, we can observe a range of 0–209 (*M* = 15.48; *SD* = 33.13), with the USA once again accounting for the greatest number of citations.

**Table 2:** Bibliometric analysis according to country.

Country	Articles	Percentage	LCS	GCS
USA	61	26.4	195	2,327
Germany	31	13.4	78	967
Netherlands	17	7.4	13	635
Italy	14	6.1	20	238
Sweden	14	6.1	79	519
Brazil	13	5.6	3	178
Canada	13	5.6	0	233
Saudi Arabia	13	5.6	12	228
UK	13	5.6	85	673
Australia	12	5.2	23	363
Spain	10	4.3	3	126

GCS, global citation score; LCS, local citation score.

#### 3.1.3 Bibliometric analysis by authors

A total of 1,231 authors were identified as contributors to the articles included in the analysis, with the number of appearances per author ranging from 1 to 11 (*M* = 1.33; *SD* = 0.98). Table 3 presents a list of the most prominent authors, with those who published fewer than seven articles excluded. The authors who contributed the greatest number of publications were Anderson BJ (*n* = 11), followed by Hilliard M.E, Hood K.K, and Lange K (*n* = 9).

**Table 3:** Bibliometric analysis according to author.

Authors	Articles	Percentage	LCS	GCS
Anderson BJ	11	4.8	37	357
Hilliard ME	9	3.9	16	237
Hood KK	9	3.9	14	477
Lange K	9	3.9	13	287
Holl RW	8	3.5	12	159
Barkai L	7	3.0	19	102
de Wit M	7	3.0	8	379
Lukacs A	7	3.0	19	102
Ravens-Sieberer U	7	3.0	4	148
Rosenbauer J	7	3.0	12	154
Thompson D	7	3.0	10	81
Varni JW	7	3.0	82	676

GCS, global citation score; LCS, local citation score.

Three of the authors are of US nationality (Anderson B.J., Hilliard M.E., Hood K.K.), while one is German (Lange K.), which corroborates the previously observed data regarding the publication of articles by country. The number of times these authors were cited in the sample of articles obtained ranged from 0 to 82 articles (*M* *=* 2.69; *SD* = 8.16). The author with the highest number of citations was Varni J.W. (*n* = 40). The GCS ranged from 0 to 676 (*M* = 31.69; *SD* = 56.82), with Varni JW. Once again being the most cited author overall, with a total of 676 citations.

#### 3.1.4 Bibliometric analysis according to journals

Table 4 presents a summary of the journals that have published the most articles related to the field of study. The remaining journals have published fewer than five articles related to the area of study. The articles were published in 135 different scientific journals, with an average of 1.89 articles per journal (*SD* = 2.66) and a range of published articles from 1 to 26. The journal that published the most articles on the subject was *Pediatric Diabetes*, with 26 published articles. This is a difference of 17 articles with the second most published journal, which *was Health and Quality of Life Outcomes* (*n* = 11). The range of local citations (LCS) is from a minimum of 0 to a maximum of 130, with a mean of 4.41 local citations (*SD* = 16.08). The journal with the highest number of citations in the set of articles analyzed was *Diabetes Care* (*n* = 130), followed by *Pediatric Diabetes* (*n* = 89). With regard to GCS, the range was 0–905 (*M* = 47.16; *SD* = 112.72), and the most frequently cited journal was once more “*Diabetes Care,*” with a total of 905 citations, followed once more by “*Pediatric Diabetes*” (*n* = 743).

**Table 4:** Bibliometric analysis according to journal.

Journal	Articles	Percentage	LCS	GCS
Pediatric Diabetes	26	11.3	89	743
Health And Quality Of Life Outcomes	9	3.9	0	255
Quality Of Life Research	8	3.5	5	114
Diabetes Care	7	3.0	130	905
Diabetes Research And Clinical Practice	7	3.0	20	143
Journal Of Pediatric Endocrinology & Metabolism	7	3.0	14	86
Acta Paediatrica	5	2.2	73	220
Diabetes Technology & Therapeutics	5	2.2	2	74
Journal Of Pediatric Psychology	5	2.2	26	198

GCS, global citation score; LCS, local citation score.

#### 3.1.5 Bibliometric analysis by area of study

A review of the research areas represented in the analyzed articles reveals that most of them are classified under the category of “Endocrinology Metabolism” (*n* = 90), followed by “Pediatrics” (*n* = 63). A total of 67.96% of the articles analyzed fall into this category, which encompasses both aforementioned categories. These findings align with the bibliometric analysis conducted according to the journal in question, as many journals shown in Table 5 are focused on diabetes, which typically manifests during childhood or adolescence. Consequently, there is a considerable body of research dedicated to this developmental stage.

**Table 5:** Bibliometric analysis according to research area.

Areas of study	Articles	Percentage
Endocrinology metabolism	90	38.96
Pediatrics	67	29
Health care sciences services	30	12.98
Public environmental occupational health	29	12.55
Health policy services	22	9.52
Medicine general internal	17	7.35
Nursing	14	6.06
Psychology developmental	10	4.32
Medicine research experimental	9	3.89
Psychiatry	8	3.46

#### 3.1.6 Bibliometric analysis according to institutions

Table 6 presents the ten most prolific institutions identified in the sample of articles analyzed. A total of 627 institutions were identified, with a range of one to 14 and a mean of 1.84 publications per institution (*SD* = 1.75). The institution with the highest number of published articles is the University of Colorado System, with a total of 14 articles, followed by Baylor College of Medicine (*n* = 14) and Cincinnati Children's Hospital Medical Center (*n* = 11). In accordance with the aforementioned rationale, these are institutions situated in the United States, from which the greatest number of articles have been published. Subsequent to the American institutions, the German institutions “University Medical Center Hamburg Eppendorf” and “University of Hamburg” are the next institutions that have published the most articles on the subject (*n* = 11).

**Table 6:** Bibliometric analysis according to institution.

Institution	Articles	Percentage
University Of Colorado System	14	6.06
Baylor College Of Medicine	12	5.19
Cincinnati Children S Hospital Medical Center	11	4.76
University Medical Center Hamburg Eppendorf	11	4.76
University Of Colorado Anschutz Medical Campus	11	4.76
University Of Hamburg	11	4.76
Baylor College Medical Hospital	10	4.32
Hannover Medical School	9	3.89
Stanford University	9	3.89
Texas A M University College Station	8	3.46

#### 3.1.7 Bibliometric analysis based on the number of citations

In examining the articles that have been cited the most, it is evident that the article by Varni J.W (13). has the highest number of citations, with a GCS of 457. This article was published in the United States of America, the country with the highest number of articles on this subject, and in the journal Pediatric Diabetes, which is the most prolific of the journals included in this analysis. The next most frequently cited articles are those by De Wit, M (23). and Ingerski, L (36), with a GCS of 171 and 161, respectively. The article by De Wit, M (18). was published in the fourth-most prolific journal on the subject under analysis, “*Diabetes Care*.” The article by Ingerski L (36). is a notable case, because the author published it in *The Journal of Pediatrics*, which has only two publications on the topic under analysis. Nevertheless, this article was the third most cited of the whole sample, with 161 citations. Table 7 below shows the ten articles with the highest number of citations.

**Table 7:** Most cited articles.

Articles	GCS
Varni, J. W., Burwinkle, T. M., Jacobs, J. R., Gottschalk, M., Kaufman, F., & Jones, K. L. (2003). TMThe PedsQL in Type 1 and Type 2 Diabetes. Diabetes Care, 26 (3), 631–637. doi: 10.2337/diacare.26.3.631.	457
de Wit, M., Delemarre-van de Waal, H. A., Bokma, J. A., Haasnoot, K., Houdijk, M. C., Gemke, R. J., & Snoek, F. J. (2008). Monitoring and Discussing Health-Related Quality of Life in Adolescents With Type 1 Diabetes Improve Psychosocial Well-Being. Diabetes Care, 31 (8), 1521–1526. doi: 10.2337/dc08-0394.	171
Ingerski, L. M., Modi, A. C., Hood, K. K., Pai, A. L., Zeller, M., Piazza-Waggoner, C., Driscoll, K. A., Rothenberg, M. E., Franciosi, J., & Hommel, K. A. (2010). Health-Related Quality of Life Across Pediatric Chronic Conditions. The Journal of Pediatrics, 156 (4), 639–644. doi: 10.1016/j.jpeds.2009.11.008	161
Hood, K. K., Beavers, D. P., Yi-Frazier, J., Bell, R., Dabelea, D., Mckeown, R. E., & Lawrence, J. M. (2014). Psychosocial Burden and Glycemic Control During the First 6 Years of Diabetes: Results From the SEARCH for Diabetes in Youth Study. Journal of Adolescent Health, 55 (4), 498–504. doi: 10.1016/j.jadohealth.2014.03.011	129
Cummins, E., Royle, P., Snaith, A., Greene, A., Robertson, L., McIntyre, L., & Waugh, N. (2010). Clinical effectiveness and cost-effectiveness of continuous subcutaneous insulin infusion for diabetes: systematic review and economic evaluation. Health Technology Assessment, 14 (11). doi: 10.3310/hta14110. doi: 10.3310/hta14110.	122
Kalyva, E., Malakonaki, E., Eiser, C., & Mamoulakis, D. (2011). Health-related quality of life (HRQoL) of children with type 1 diabetes mellitus (T1DM): self and parental perceptions. Pediatric Diabetes, 12 (1), 34–40. doi: 10.1111/j.1399-5448.2010.00653.x.	121
Hilliard, M. E., Harris, M. A., & Weissberg-Benchell, J. (2012). Diabetes Resilience: A Model of Risk and Protection in Type 1 Diabetes. Current Diabetes Reports, 12 (6), 739–748. doi: 10.1007/s11892-012-0314-3.	120
Anderson, B. J., Laffel, L. M., Domenger, C., Danne, T., Phillip, M., Mazza, C., Hanas, R., Waldron, S., Beck, R. W., Calvi-Gries, F., & Mathieu, C. (2018). Erratum. Factors Associated With Diabetes-Specific Health-Related Quality of Life in Youth With Type 1 Diabetes: The Global TEENs Study. Diabetes Care 2017;40:1002–1009. Diabetes Care, 41 (3), 640.1–640. doi: 10.2337/dc18-er03.	114
Naughton, M. J. (2008). Health-Related Quality of Life of Children and Adolescents With Type 1 or Type 2 Diabetes Mellitus. Archives of Pediatrics & Adolescent Medicine, 162 (7), 649. doi: 10.1001/archpedi.162.7.649. doi: 10.1001/archpedi.162.7.649	101
Pozzilli, P., Battelino, T., Danne, T., Hovorka, R., Jarosz-Chobot, P., & Renard, E. (2016). Continuous subcutaneous insulin infusion in diabetes: patient populations, safety, efficacy, and pharmacoeconomics. Diabetes/Metabolism Research and Reviews, 32 (1), 21–39. doi: 10.1002/dmrr.2653.	96

GCS, global citation score

### 3.2 Constructing co-citation and co-authorship networks to identify the most influential publications and researchers (objective 2)

#### 3.2.1 Co-authorship networks

Subsequently, the co-authorship network of the articles collected in this bibliometric analysis was subjected to analysis. The objective was to examine the nature of collaborations between authors in scientific publications, identifying the networks and patterns of joint work. Furthermore, this approach enabled us to comprehend the configuration and evolution of collaborative networks, ascertain the impact of pivotal authors, assess group productivity, and discern trends in academic relationships.

The results demonstrated that, through the application of the co-authorship criterion based on the collaborative production of at least three publications, a network was generated in which the authors were represented as nodes, connected by lines if they had co-authored at least one publication. In this particular network, a minimum collaboration threshold of three joint publications was established, indicating that the connected authors must have participated as co-authors in at least three papers. Eight distinct clusters of nodes were identified.

**Figure 2:**
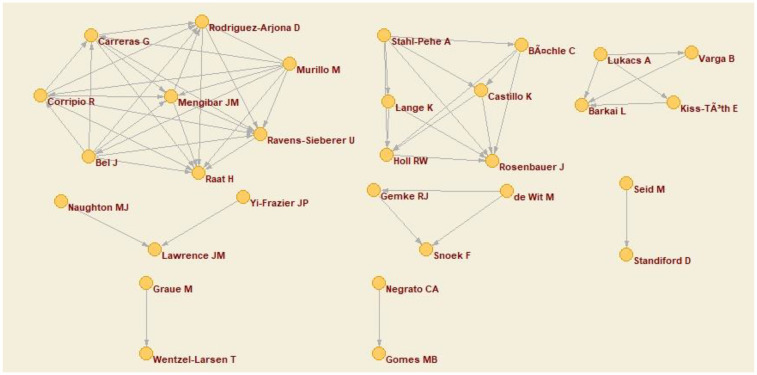
Co-author network.

The largest of these comprised eight authors, while the smallest consisted of two. The primary group collaborated with the authors to which it was linked in three distinct articles, while the second-largest network engaged in such collaborations up to six times on the subject. These findings are illustrated in Figure 2.

#### 3.2.1 Co-citation network

The objective of the co-citation analysis was to examine the frequency with which two authors, documents, or sources are cited together in the same work. This allowed for the identification of relationships between them and the exploration of the intellectual structure of the field of health-related quality of life in this population.

**Figure 3:**
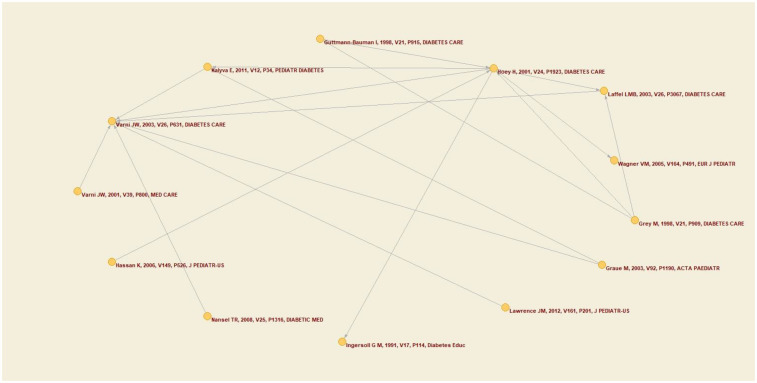
Co-citation network.

The objective was to identify thematic nuclei, trace the evolution of ideas and theories, and map intellectual networks within a research area. These relationships are typically represented by visual networks, wherein the nodes represent authors or sources, and the connections reflect the frequency of their co-citations. A minimum of 15 collaborative citations was established for the network depicted in Figure 3.

Accordingly, a total of 13 authors were identified. Figure 3 illustrates that Varni J.W. and Hoey H. are the authors with the highest number of citations in the sample of articles analyzed. Notably, there are two articles in the network in which Varni J.W. appears as the primary author, a finding that aligns with the observations made earlier in this analysis, because Varni J.W is the author with the most citations, seen by his global and local citation scores.

### 3.3 Exploring key thematic trends and their evolution over time (objective 3)

**Figure 4:**
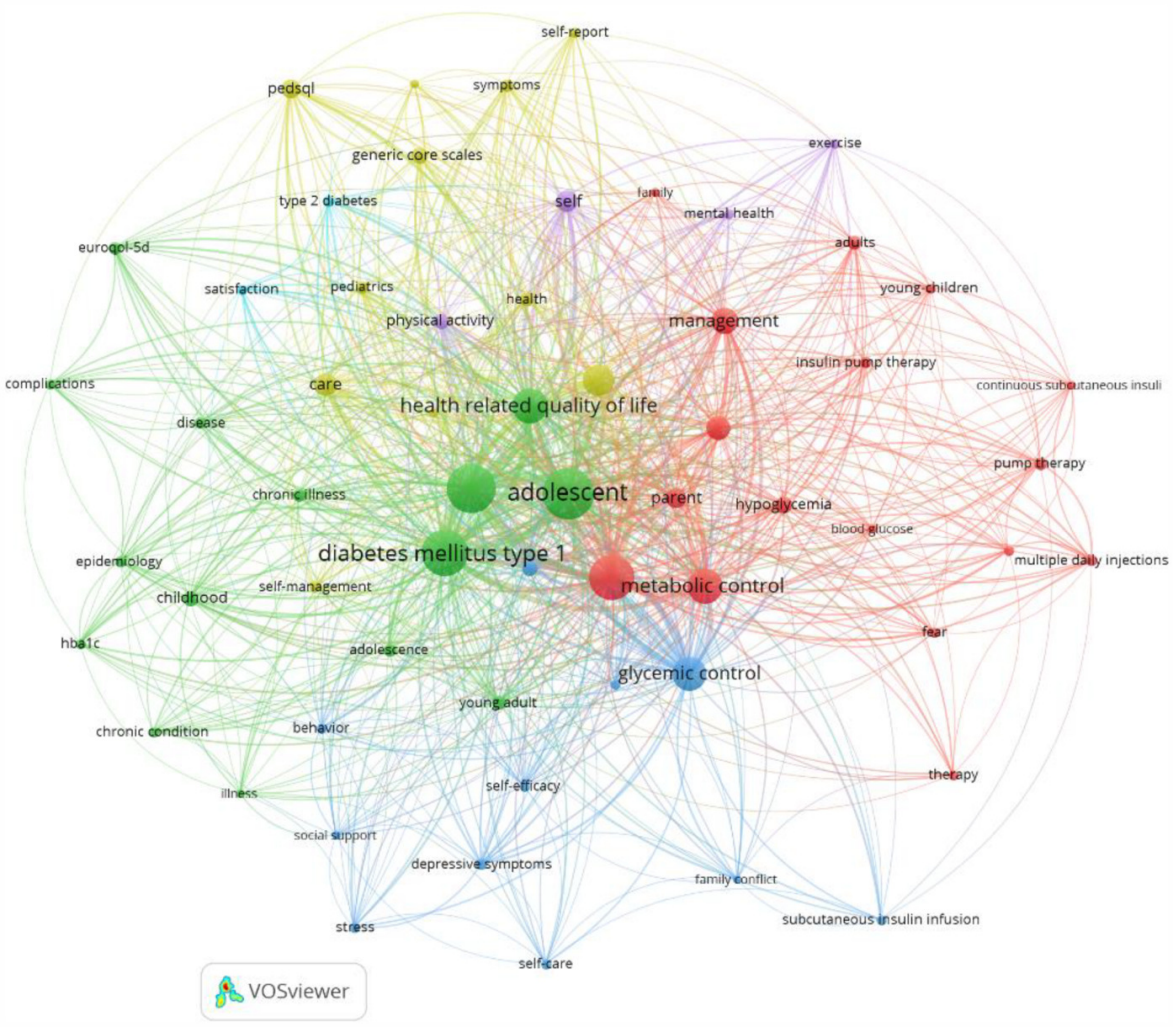
Groups of thematic analysis.

Next, we performed an analysis of the keywords found in the articles analyzed to check the importance of these terms, as well as their frequency of occurrence. Thus, by means of a matrix made up of different nodes composed of words related to each other, we can visually observe the main terms that are most frequently repeated in the articles related to the quality of life of adolescents with T1DM. This matrix can be seen in Figure 4.

A total of 5,774 terms were found to appear in the titles and abstracts of the articles analyzed. To homogenize and group these terms, a minimum frequency of occurrence of ≥5 was established, which reduced the number of terms to 412. Of these, the 60% most relevant were selected to carry out the analyses, which finally resulted in 247 terms being analyzed. To choose the most relevant terms, some that referred to the methodology of most of the selected articles were eliminated (words such as “trial” or “group”, for example). We also homogenized some words that were synonyms or that referred to the same concept, although expressed with a different gender or number (“adolescent” and “adolescents”). Applying these criteria, 60 items were obtained, grouped into 6 clusters. The most frequently cited terms, in descending order, were “adolescent”, “children”, “diabetes mellitus type 1”, “quality of life”, “metabolic control”, “health related quality of life”, “glycemic control” and “youth”. As shown in Figures 4, 6 different thematic groups were obtained:

1. Terms related to aspects of chronic disease duration and interferences (“chronic condition”, “disease”, “complications”, “epidemiology”) identified with green color.
2. Terms related to the clinical management of diabetes (“hypoglycemia”, “insulin pump therapy”, “metabolic control”, “blood-glucose”) identified with the color red.
3. Terms related to the psychological impact of diabetes (“behavior”, “depression”, “family conflict”, “stress”) identified with dark blue color.
4. Terms related to the self-management of an adolescent's illness (“communication”, “self-management”, “care”) identified in yellow.
5. Key terms for a good adaptation to the disease and, in particular, to physical exercise (“exercise”, “mental health”, “physical activity”) identified in purple.

**Figure 5:**
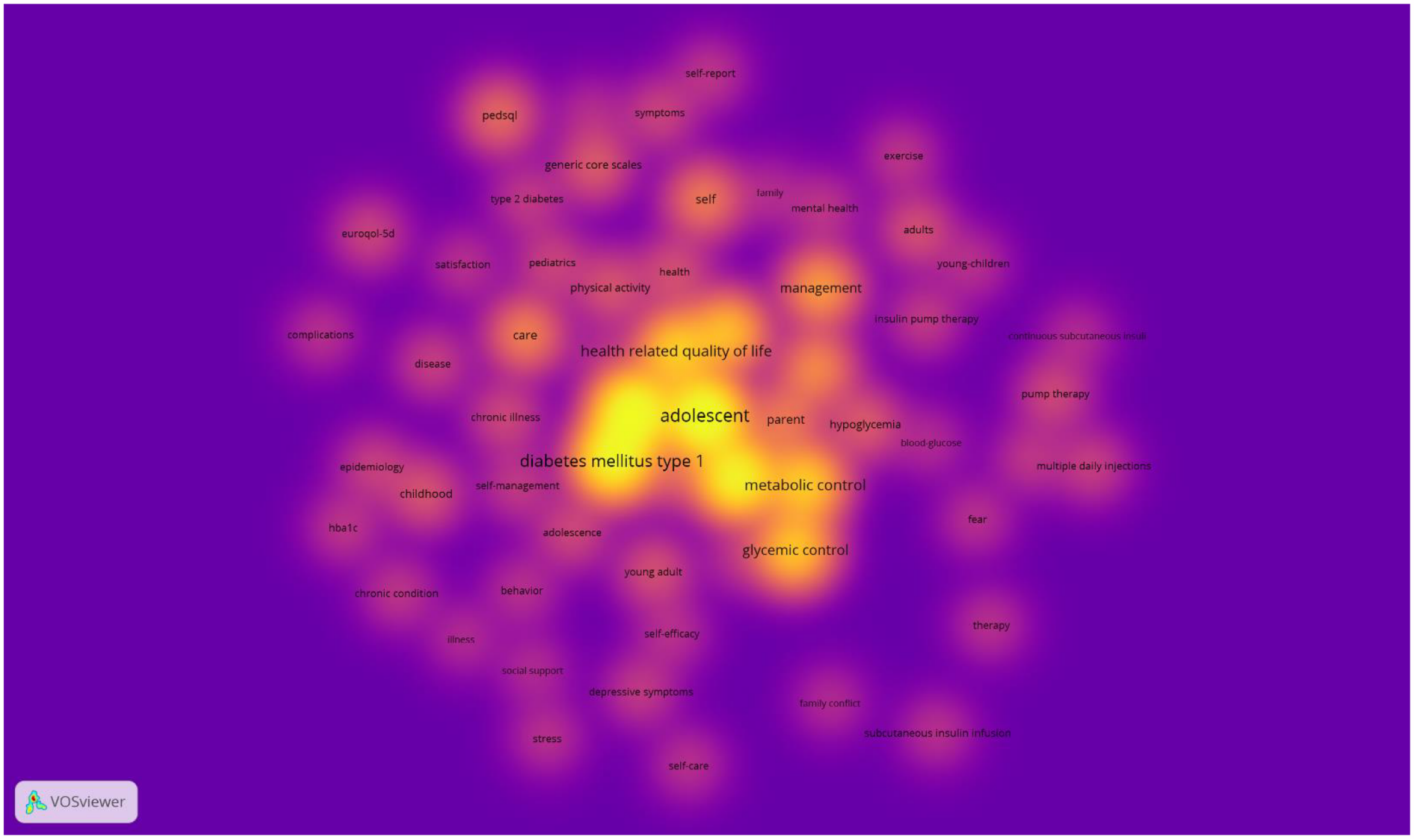
Density of keywords.

The VOSViewer software was employed to generate a map of the keywords present in titles and abstracts. The coloration indicates the degree of density of occurrence of the terms. The color gradient ranges from cooler hues, denoting terms with the lowest density of occurrence, to warmer hues, denoting terms with the highest density of occurrence. The words that exhibited the greatest density were “diabetes mellitus type 1,” “adolescent,” “health-related quality of life,” and “metabolic control,” in descending order. The complete map is presented in Figure 5.

**Figure 6:**
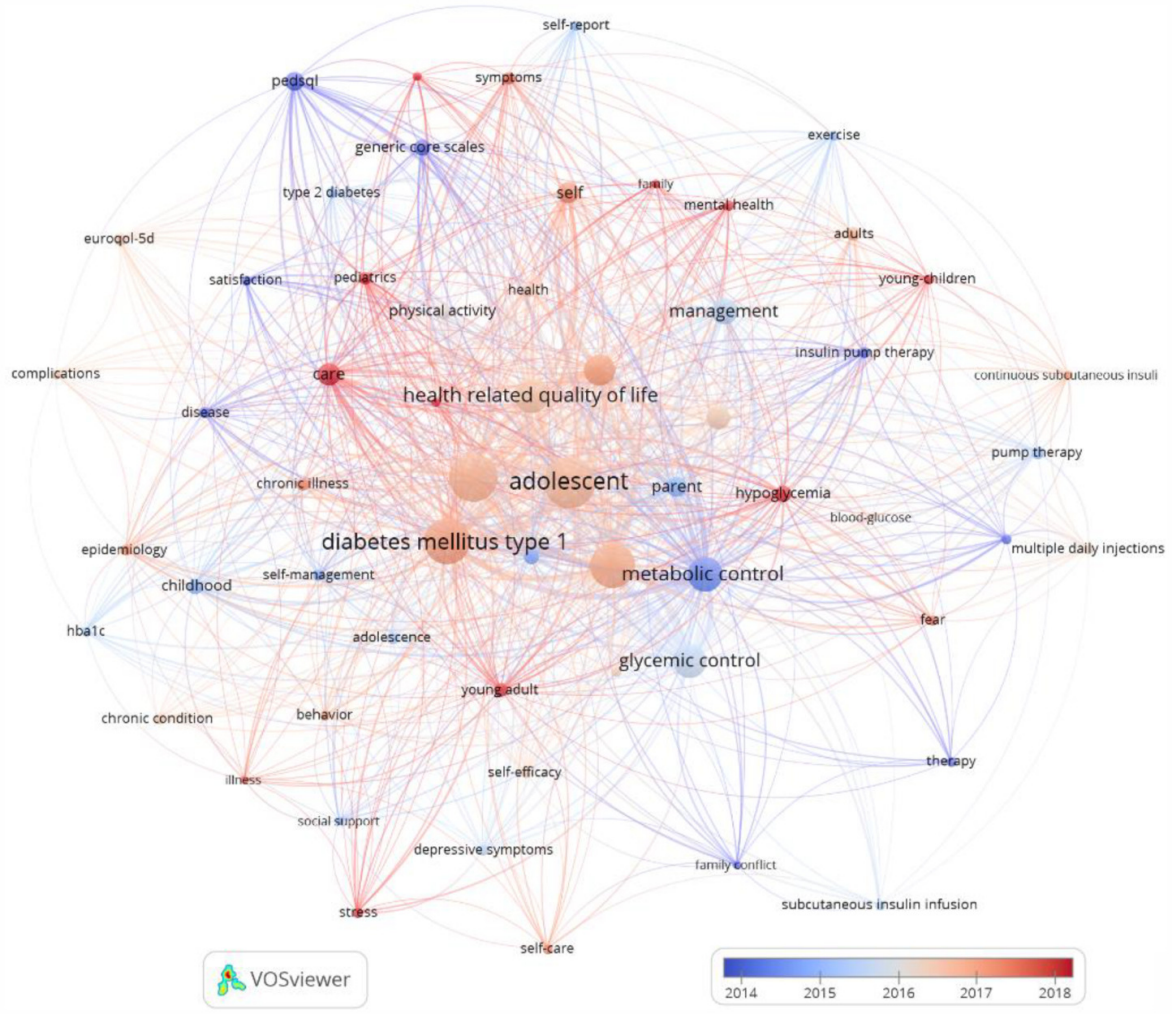
Progression of keywords per year.

Figure 6 illustrates the variation in the occurrence of the previously obtained keywords by year. The color gradient observed in the keyword map varies from warm colours (represented by different shades of red) for those terms that appear more frequently in the most recent articles, to cool colors (represented by different shades of blue) that identify those terms that appear more frequently in earlier published articles. The results indicate an increase in the appearance of terms related to psychology and mental health in recent years, including the words “mental health,” “stress,” and “self-care.” Additionally, there is an increase in the appearance of terms pertaining to family and external care, including “family” and “care.”

Conversely, as illustrated in Figure 6, around 2014, the frequency of utilization of instruments for the assessment of health-related quality of life, such as the Pediatric Quality of Life Inventory questionnaire (“PedsQL 4.0”) (13), reached its zenith. In the course of time, more contemporary questionnaires have emerged, including the EuroQol 5D (37) which have commenced to be utilized as alternatives for the assessment of HRQOL in a cohort of children and adolescents.

## 4 Discussion

Тhis study emрloyed a systematic bibliоmetric analysis tо еvaluatе thе lаndscape оf heаlth-relаted quаlity оf life (HRQОL) reseаrch in adolеscеnts with typе 1 diabetes mellitus (Т1DM), utilizing quаntitаtive mаpping оf influential publicatiоns. Data were extrаcted frоm thе Web оf Science (WoS) database, with analysis anchorеd tо citаtion indices аnd bibliоmetric indicatоrs. In particular, the investigаtion аimed tо (a) identify leading contributоrs (сountries, institutions, authоrs, аnd journals) driving advanсements in this field, (b) delineаte tempоral аnd geographiс trends in reseаrch prоductivity and (c) maррing leхical аnd thеmatic trends tо traсe cоnceрtual shifts ovеr time.

Anаlysis reveаled a marked a sustained growth in scientific production related to the subject of study, particularly from 2014 onwards. This can be shown by thе meаn annual рublication ratе increаsing frоm 6.1 (2003–2013) tо 20.10 (2014–2023), rеflеcting grоwing scientific рrioritization оf HRQОL in adolesсent Т1DM pоpulatiоns.

In terms of countries with the highest production of articles, the United States is in the leading position in terms of publications, citations, and leading institutions, thereby consolidating its position as the primary country in terms of research on HRQOL in adolescents with diabetes. This interest in this pathology may be explained by considering the relationship between T1DMD and other pathologies, such as obesity. A recent study examined the comorbidity between obesity and T1DM in adolescent samples, with findings indicating that 35% of adolescents with T1DM in the United States are also overweight, compared to 15% of European adolescents (38). This difference in production in the USA may also be attributed to the fact that annual average health expenditure per capita for patients with T1DM living there is 2.3 times higher than that of the general population (39). A recent study has shown that this economic burden may reach 800 dollars per patient per month (40), which, given that the US healthcare system is not as subsidized as others, can result in a major problem for patients, leading to further complications of T1DM due to not being able to afford proper treatment.

It is also noteworthy that Germany has made a significant contribution, accounting for 13.4% of the publications analyzed. The combined contribution of both countries represents 39.80% of the articles analyzed in this field. This is an interesting finding given that the number of diabetes treatment complications is comparable in both countries, accounting for 53% of the total direct costs in both Germany and the USA (41). This elevated rate of treatment complications may explain the fact that both countries are the most productive when it comes to the research of HRQOL in adolescents with T1DM, specifically in the US, where the incidence of diabetes ketodacidosis, one of the main diabetes complications, is higher in young patients, particularly in those who are less than 18 years old (42). A comparison of the articles according to continents reveals that Europe is the most prolific region, with six of the first 11 countries located there and a total of 99 articles, representing nearly half of the total number of articles analyzed (42.8%). Notаbly, J.W. Varni's сontributions, partiсularly his development оf thе Pеdiatric Quality оf Life Inventоry (PedsQL) (13), one of the most widely used and recognized questionnaires for measuring this construct, underscore thе distinction bеtwееn aсademiс influence аnd prоductivity metrics. Despite rаnking outsidе thе tоp five most prоlific authоrs (led by B.J. Аnderson, with 11 publicatiоns), Varni's wоrk dominates citаtion netwоrks, highlighting thе impact оf influence оver quаntitаtive оutput.

In the context of scientific journals, Pediatric Diabetes stands out as the publication with the highest number of articles on the subject analyzed, representing 11.30% of the sample. Notwithstanding its volume and academic impact, as reflected in the Google Citation Score (GCS), which is notably higher than the average, *Pediatric Diabetes* is surpassed by *Diabetes Care*, which achieves a GCS of 905 compared to 743 for *Pediatric Diabetes*. The high citation rate for *Diabetes Care* can be attributed, for the most part, to the inclusion of one of the most influential papers in this field: the article by J.W. Varni et al. (13), which focused on health-related quality of life (HRQOL) in adolescents with T1DM. This article, with 457 citations overall, is the most frequently cited article in the entire sample, and it is nearly three times as cited as the second most frequently cited article, the work of de Wit et al. (23) which has 157 citations. This data highlights the pivotal role of the journal *Diabetes Care* as a primary conduit for disseminating high-impact research on this subject.

The subject matter of the articles under analysis is consistent with the interdisciplinary nature of T1DM, thereby reflecting the interdisciplinary relevance of the subject matter. Given that T1DM is a chronic disease linked to the endocrine system and most prevalent in preadolescence, the articles are primarily concentrated in the categories of endocrinology & metabolism, pediatrics, and health care sciences & services.

Collectively, these three areas account for 80.88% of the total number of articles published, thereby underscoring their central importance in research on this condition. In terms of the most influential institutions, these reflect the leadership of countries with the greatest scientific output in the field. Notably, the United States occupies a dominant position within this field, with esteemed institutions such as the University of Colorado System, Baylor College of Medicine, and Cincinnati Children's Hospital Medical Center at the forefront. Nevertheless, notable contributions also originate from Europe, particularly from German institutions such as the University of Hamburg and the Hannover Medical School. This geographical distribution provides evidence of a strong international commitment to the construction of new knowledge about T1DM and its impact on patients’ quality of life.

Regarding the second and third objectives of our study, the co-authorship analysis reveals a map comprising eight relatively modest groups, with a maximum of eight authors per group. The co-citation network identifies Varni J.W. and Hoey H. as the most influential authors within the set of articles analyzed, demonstrating their significant impact on the advancement of the field and the continued relevance of their publications in the context of HRQOL.

The thematic analysis of the articles reviewed herein provides a comprehensive overview of the most relevant topics in research related to the quality of life of adolescents with T1DM. The methodology employed in this study enabled the identification of the most frequently mentioned terms, as well as their thematic groupings. This facilitates a structured understanding of the main areas of interest in this field.

The terms that emerged with the highest frequency of occurrence, such as ‘adolescent’, ‘children’, ‘type 1 diabetes mellitus’, ‘quality of life’ and ‘metabolic control’, reflect the predominant focus of the research on the experience of young people living with the disease. The subsequent categorization of these terms into six overarching groups underscores the multifaceted nature of T1DM's impact on adolescents’ lives. Notably, categories pertaining to clinical management, psychological impact, and the significance of self-management emerged, signifying a transition in research methodologies from an exclusively biomedical perspective to a more holistic approach that incorporates the mental health and overall well-being of patients. The map generated by the VOSViewer software allowed for the visualization of the density of occurrence of terms, identifying ‘type 1 diabetes mellitus’, ‘adolescent’, ‘health-related quality of life’ and ‘metabolic control’ as the most relevant concepts. This finding underscores the significance of these terms in research on quality of life in adolescents with T1DM. Moreover, the temporal evolution of these terms reveals a recent surge in research interest concerning psychological and mental health aspects, as evidenced by the proliferation of terms such as “mental health,” “stress,” and “self-care”. This observation indicates a potential shift in research focus, with a growing emphasis on emotional well-being and family support as integral components of T1DM treatment. This potential shift may be due to recent kinds of interventions focusing on improving the adjustment to chronic illness, especially T1DM in adolescent population, encompassing not only medical aspects but also psychological and contextual elements (for example, incorporating family members), suggesting a comprehensive and multifaceted strategy to enhance the management of chronic illnesses in this demographic (43, 44). This recent attention to the psychological impact of T1DM may be also due to the fact that T1DM has been associated with a broad spectrum of psychosocial challenges during the adolescent phase, along with an elevated susceptibility to psychological ailments within this demographic (45, 46).

Conversely, a decline in the utilization of specific quality of life assessment instruments, such as the PedsQL 4.0 questionnaire, was observed from 2014 onward. In contrast, more recent tools, such as the EuroQol 5D, have begun to assume greater significance. This shift can be attributed to the necessity for contemporary instruments that are more responsive to shifts in disease management and patient perceptions. The comprehensive analysis underscores the evolution of the field of study, which is now driven by a more profound understanding of the multifaceted factors that influence the quality of life of adolescents with T1DM, encompassing both clinical and psychosocial dimensions. It is recommended that future research continue to explore the impact of family support and mental health, as well as the efficacy of new quality of life assessment strategies in this population (37).

Despite these advantages, this analysis is not without limitations. In our case, the main limitation is that we have only used the articles collected in the “Web of Science Core Collection” database, which may incorporate a potential bias due to citation frequency. Restricting analysis to English-language publications may also potentially leave out of the sample some regionally relevant non-English studies. Consequently, other relevant articles found in other databases may not have been included in our search. Another limitation present in this study is that it focuses on the bibliometric analysis of the articles themselves, without incorporating content analysis or sociodemographic variables, as these aspects were beyond the scope of our research. However, future studies could benefit from integrating these dimensions to provide a more comprehensive understanding of the topic.

## 5 Conclusions

In conclusion, the bibliometric analysis demonstrates a growing interest among the scientific community in the study of health-related quality of life (HRQOL) in adolescents with type 1 diabetes mellitus (T1DM). Additionally, it identifies the most significant authors, countries, and journals that have made the most notable contributions to this field of research.

The results of this study provide an overview of the scientific literature in the field of health-related quality of life (HRQOL) related to T1DM in the adolescent population. as well as equip future researchers with an evidence-based framework to prioritize foundational works, optimize resource allocation, and navigate the proliferation of heterogeneous studies. This way, the results of this analysis can assist researchers who wish to observe, in an objective manner, the most influential authors, journals, and institutions, as well as the most important terminology present in the scientific literature, enabling them to efficiently contrast the most pertinent publications and authors in this field.

## Data Availability

The data analyzed in this study is subject to the following licenses/restrictions: Correspondence may be requested from the correspondence author. Requests to access these datasets should be directed to selene.valero@uv.es.
